# Effect of Laser Shock Peening on High-Cycle Fatigue Performance and Residual Stress in DH36 Welded Joints

**DOI:** 10.3390/ma18225178

**Published:** 2025-11-14

**Authors:** Shengguan Qu, Yulian Sha, Yi Hou, Jianhua Wang, Fenglei Li, Xiaoqiang Li

**Affiliations:** 1 School of Mechanical and Automotive Engineering, South China University of Technology, Guangzhou 510640, China; 18894619540@163.com (Y.S.); yi_hou186@foxmail.com (Y.H.); flli@scut.edu.cn (F.L.); lixq@scut.edu.cn (X.L.); 2 National Engineering Research Center of Near-Net-Shape Forming for Metallic Materials, Guangzhou 510640, China; 3 Guangzhou Shipyard International Corporation Limited, Guangzhou 511462, China; wjh@chinagsi.com

**Keywords:** high-strength steel, welded joint, laser shock peening (LSP), residual stress, high-cycle fatigue, fatigue limit, microhardness

## Abstract

DH36 high-strength steel is widely used in shipbuilding and other fields due to its excellent strength, low-temperature toughness, wear resistance, and corrosion resistance. However, the harsh deep-sea environment seriously reduces the service life of welds. In this study we subjected DH36 welded joints to laser shock peening at three different energy levels (5 J, 7 J, 9 J) to investigate its effects on microhardness, microstructure, high-cycle fatigue, and residual stress of the DH36 welded joints. Results indicate that LSP can significantly enhance the surface microhardness of welded joints. Notably, the 7 J energy treatment increased the weld zone microhardness from 195 HV_0.2_ to 231 HV_0.2_ (18.5% improvement) and the heat-affected zone microhardness from 194 HV_0.2_ to 234 HV_0.2_ (20.6% improvement). Residual tensile stress on the specimen surface was offset and replaced by residual compressive stress after LSP. Concurrently, the high-cycle fatigue limit of the specimens was significantly improved, with the most pronounced improvement observed in specimens subjected to 5 J energy—increasing from 258 MPa to 295 MPa, representing an increase of 14.34%.

## 1. Introduction

At present, the economies of countries around the world are in a stage of continuous development, and the demand for energy is constantly increasing, making the development of clean and renewable energy increasingly urgent. The ocean possesses enormous renewable energy resources, and the related marine energy technologies and equipment have attracted widespread attention [1]. To access greater energy resources, marine activities are gradually expanding from nearshore to deep-sea regions. Harsh operating environments pose greater challenges to vessels, requiring marine equipment materials to meet the demanding conditions of deep-sea operations [2]. DH36 is a high-strength ship plate steel characterized by excellent weldability, high strength, good low-temperature toughness, and superior wear and corrosion resistance. It exhibits extended service life in harsh marine environments and finds extensive application in shipbuilding and related fields [3,4]. The United States has incorporated DH36 into its “Lightweighting Innovation Program”, establishing a temperature-related performance database and optimizing welding processes to enhance deformation resistance under extreme deep-sea conditions [5]. China employs DH36 as a core material in deep-sea wind power structures. Currently, DH36 high-strength steel is utilized in Fujian’s ZhangPu Liuao Offshore Wind Farm Phase II, Shandong’s Huaneng Shandong Peninsula L-Field Offshore Wind Farm.

In deep-sea vessels, steel components are to be connected through welding. The lifespan of welds critically impacts marine vessel safety and operational performance. During welding, differences of heat input and cooling rate across sections cause uneven expansion and contraction in welded structures, which induces internal residual stress and microstructural inhomogeneity, seriously affecting the fatigue life and reliability of the welded structure [6,7]. Residual tensile stress from welding reduces structural strength, accelerates crack initiation and propagation, and shortens deep-sea vessels’ fatigue life [8,9]. Uneven deformation from welding increases assembly errors and degrades structural precision [10]. Furthermore, when vessels operate in deep-sea environments, they are also subjected to the coupling effect of multiple factors: for example, high pressure increases weld porosity; low temperature exacerbates weld hardening and embrittlement; seawater corrosion, wave dynamic loads compounded by sea winds, and equipment vibration loads will promote crack propagation and reduce fatigue life. All these factors will seriously shorten welded structures’ service life [11,12]. Therefore, it is crucial to strengthen the welds to maintain excellent performance in deep-sea environments.

Laser Shock Peening (LSP) is a surface strengthening technique that refines the surface layer grains to the submicron or even nanometer scale and induces residual compressive stress, thereby achieving strengthening effects. LSP employs short-pulse (nanosecond, picosecond, or even femtosecond) high-power (GW/cm^2^) laser beams directed at metal surfaces. The absorber layer coated on the metal surface absorbs laser energy and generates a large amount of dense high-temperature (>10^7^ K) and high-pressure (>GPa) plasma. The plasma continues absorbing laser energy, rapidly expanding outward and undergoing explosive vaporization to form a laser shock wave. When plasma pressure exceeds the material’s dynamic yield limit, irreversible plastic deformation occurs [13]. Consequently, LSP leads to the formation of a fine-grained structure on the surface of the steel, resulting in increased residual compressive stress, hardness and strength [14,15]. Thus, LSP can improve the wear resistance and fatigue properties of materials, which is of great engineering significance in practice [16]. Compared to shot peening and surface ultrasonic rolling techniques, LSP has the advantages of higher strain rate, deeper residual stress layer, and more precise process parameters and strengthened areas [17].

Numerous scholars have explored the strengthening mechanisms of the LSP process from multiple perspectives, including microstructure, microhardness, and residual stress. Pan et al. [18] subjected thin-walled AA7075 aluminum alloy specimens to low-pulse LSP strengthening and analyzed the effect of LSP on fatigue performance by residual stress and microstructure. The study found that after LSP, the surface layer of the specimens formed heterogeneous grains composed of ultrafine and coarse grains and the residual compressive stress exhibited high cyclic stability. Both factors effectively suppressed fatigue crack propagation. LSP treatment at a lower laser power density (1.8 GW/cm^2^) improved fatigue performance, increasing the high-cycle fatigue limit by 20.4% for smooth specimens and 37.0% for pre-cracked specimens. Yang et al. [19] investigated the effect of LSP on the fatigue performance of 25Cr2Ni4MoV alloy. Through experimental and simulated fatigue tests, they found that the fatigue striation spacing decreased from 1.34 μm to 1.03 μm after strengthening, with the fatigue life of specimens increasing by up to 74.9%.

Many researchers have applied LSP technology to weld strengthening, exploring its mechanism for enhancing weld fatigue life. Zhou et al. [20] analyzed dissimilar welded joints of 1Cr18Ni9Ti austenitic stainless steel and GH1140 nickel-based high-temperature alloy, investigating LSP’s effects on residual stress, microhardness, microstructure, and high-cycle fatigue performance. The study revealed that LSP transformed residual tensile stress in the weld and heat-affected zone into high-amplitude residual compressive stress; the hardness of the welded joints is increased, with the hardness of the weld zone increased from 162 HV to 229 HV (an increase of 41.4%); the high-cycle fatigue limit is increased from 289 MPa to 478 MPa (an increase of 65.39%). Li et al. [21] conducted comparative experiments on 7075 aluminum alloy welded joints using three distinct parameter sets: non-strengthened (N-LSP, 0 times), low-energy LSP strengthening (L-LSP, 70 times), and high-energy LSP strengthening (H-LSP, 130 times). The study found that the surface microhardness of the specimens was significantly increased, from 121.5 HV (N-LSP) to 133.7 HV (L-LSP) and 145.2 HV (H-LSP); the proportion of high grain boundary angles in H-LSP increased, with the weld zone and heat-affected zone ratios increasing from 65.67% and 90.87% to 91.89% and 98.97%, respectively; the ultimate tensile stress increased from 282.64 MPa (N-LSP) to 320.54 MPa (L-LSP) and 337.24 MPa (H-LSP); fatigue resistance also showed significant improvement. Shi et al. [22] investigated the effects of laser quenching and LSP on the wear and fatigue resistance of field-welded rail joints through rolling contact fatigue tests, finding that the LSP process simultaneously enhanced both wear and fatigue resistance of the welded joints.

In this study, LSP was applied to the DH36 welds of offshore wind turbines to explore the strengthening effect of LSP on welds. DH36 steel welded plates were fabricated using flux-cored arc welding. The study investigated the influence of LSP on the microhardness and surface morphology of the welded joints. Fatigue limit was determined through tensile-compression fatigue testing. Additionally, numerical simulations were performed for the welding and LSP processes to analyze the residual stress of the welded joints, thereby evaluating LSP’s impact on the high-cycle fatigue performance of DH36 welded joints.

## 2. Experimental Methods

### 2.1. Materials Preparation and FCAW Process

Flux-cored Arc Welding (FCAW) was employed to perform horizontal butt welding on two DH36 steel plates, each measuring 300 mm × 100 mm × 8 mm. YCJ501-1 filler wire(Wuhan Tiemao Factory, Wuhan, China) with a diameter of 1.2 mm was used, and the chemical compositions of the filler wire and base metal are shown in Table 1. Tensile tests were conducted on the DH36 base metal, yielding the mechanical properties listed in Table 2. Before welding, the plate to be welded was cleaned with acetone to remove surface dirt and impurities. Following this, the grooves were prepared, and tack welding was completed with appropriate pre-set reverse distortion. The welding process employed CO_2_ as the shielding gas and utilized a three-pass procedure: first completing the root pass in the center, followed by the fill pass on the front side, and finally the cover pass on the reverse side. The welding current was set at 250 A, the welding voltage at 30 V, the welding speed for the root and cover passes at 500 mm/min, and the welding speed for the fill pass at 300 mm/min.

**Figure 1:**
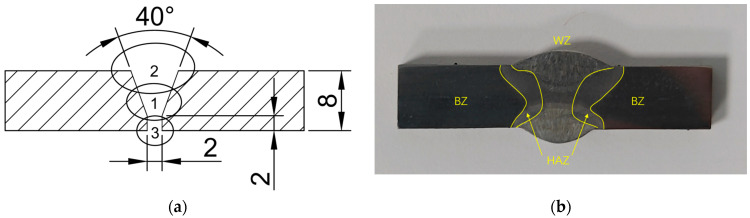
DH36 Welded Joint. (**a**) Groove configuration (**b**) Weld cross-section.

The groove configuration and welding sequence are depicted in Figure 1a, the serial numbers “1”, “2”, and “3” in Figure 1a indicate the sequence of the weld layers, and the corresponding cross-section of the welded joint is shown in Figure 1b. Macroscopic observation of the welded joint cross-section via Figure 1b clearly reveals that there are no typical internal defects such as porosity, inclusions, unfused, cracks, etc. inside the welded joints, and the whole is relatively homogeneous. Based on microstructural characteristics in Figure 1b, the entire welded joint consists of three distinct regions: the welding zone (WZ), the heat-affected zone (HAZ), and the base zone (BZ).

### 2.2. Laser Shock Process (LSP)

**Figure 2:**
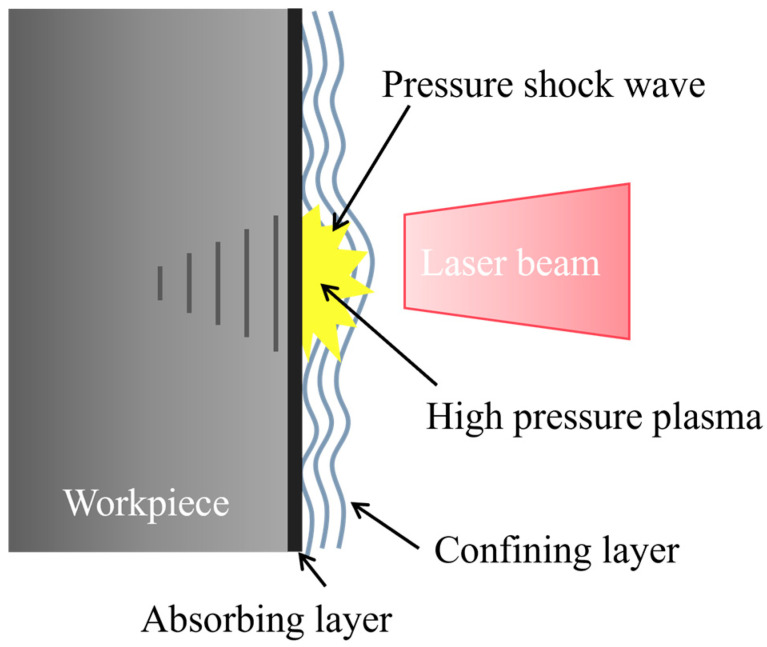
Schematic diagram of LSP principle.

LSP is a surface modification technology that enhances the performance of DH36 welded joints through the process of “energy conversion–plastic deformation–synergistic strengthening” with its strengthening mechanism illustrated in Figure 2. A high-intensity pulsed laser acts on the absorbing layer on the specimen surface. The absorbing layer absorbs the laser energy and generates a large amount of plasma. Since there is a confining layer outside the absorbing layer, the laser-induced shock wave formed after plasma expansion propagates into the material. When the peak pressure of the shock wave exceeds the yield strength of the material, the material undergoes plastic deformation. A residual compressive stress layer is formed inside the material after LSP. Concurrently, plastic deformation under high strain rates causes grain fragmentation and recrystallization, leading to a great increase in dislocation density. The residual compressive stress induced by LSP can effectively neutralize the residual tensile stress in DH36 welded joints. Grain refinement substantially enhances the hardness of the welded joints, thereby delaying the fatigue crack expansion, and extending the fatigue life of the offshore wind turbine welds.

Based on the empirical laser power density formula proposed by Fabbro et al. [23] and combined with the yield strength of DH36 steel, it is known that laser energy exceeding 4.43 J induces plastic deformation on the material surface. When laser energy reaches 11 J, plastic deformation reaches saturation. Therefore, three distinct laser energies were set for this study: 5 J, 7 J, and 9 J. Black tape was employed on the material surface as the absorber layer to protect the specimen surface from laser ablation, while thin and uniform flowing water covering the tape as the confinement layer to restrain the plasma expansion. The spot diameter was set at 3 mm. To achieve a more uniform material surface, an overlap rate of 50% was adopted [24]. Detailed LSP process parameters are presented in Table 3, where LSP1, LSP2, and LSP3 represent the LSP specimens with laser energies of 5 J, 7 J, and 9 J, respectively. The original unstrengthened specimen was recorded as N-LSP. In this study, a laser shock peening device (YS1505-R200EA Tyrida, Xi’an, China) was employed, featuring a pulse width of 20 ns and a wavelength of 1064 nm.

**Figure 3:**
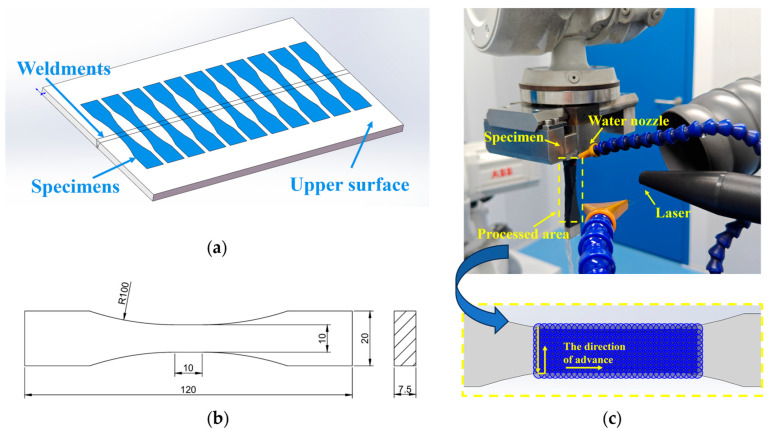
(**a**) Schematic diagram of FCAW welding (**b**) Schematic diagram of tensile-compressive fatigue dimensions (**c**) Schematic diagram of LSP strengthening and strengthening path.

To simultaneously meet the requirements of LSP and tensile-compression fatigue tests, the size of the LSP specimens was consistent with the size for tension-compression fatigue tests. Specimens of the required shape and size were cut from the welded plate using wire cutting. The excess weld reinforcement on the upper and lower surfaces was ground away. Subsequently, the surfaces were progressively polished using #180, #600, #1000, and #1500 grit sandpaper to minimize stress concentration caused by surface roughness. The specimen dimensions are shown in Figure 3b. The tensile-compression fatigue specimens are of a dog-bone shape with a rectangular cross-section, whose dimensions are strictly designed in accordance with GB/T 3075-2021 [25]. These dimensions are tailored to match the thickness of DH36 welded plates (8 mm) and the fixture specifications of the fatigue testing machine: the total length is 120 mm, the total width is 20 mm, the radius of the transition arc is 100 mm (to ensure smooth transition and prevent stress concentration), and the thickness is 7.5 mm (with 0.5 mm of weld reinforcement ground off). In the LSP, A zigzag path was employed to strengthen all four faces of the specimen as illustrated in Figure 3c. To minimize specimen deformation, the two side surfaces were strengthened first, and then the upper and bottom surfaces were strengthened.

### 2.3. Microhardness, Microstructure and Surface Roughness Measurements

The hardness distribution on the welded joint surface was measured using a DHV-1000Z Vickers hardness tester (Shanghai Shangcai Testing Machine Co., Ltd., Shanghai, China). Test parameters and operations strictly adhere to ASTM E92-22 [26]: the loading force is 200 g (1.96 N), with a load dwell time of 15 s to ensure indentation stability. Measurements were taken at 0.5 mm intervals, with three measurements per point averaged. The microstructure of the specimens was observed through a LEICA-M165C optical microscope (Wetzlar, Germany). Surface topography characteristics were measured by an RTEC Up S-Dual 3D optical profilometer (San Jose, CA, USA). The sensor probe traversed the target specimen surface to collect data, thereby obtaining surface roughness Sa and crater depth values.

### 2.4. High-Cycle Fatigue Tests

High-cycle fatigue tests were conducted using an INSTRON 8801 tensile-compression fatigue testing machine (Boston, MA, USA). The tension-compression fatigue test was conducted in accordance with GB/T 3075-2021: the stress ratio R = −1, frequency of 20 Hz, and the fatigue limit was set as 10^6^ cycles. Each specimen group comprised 4–5 stress levels, with 3–4 specimens tested per stress level to ensure result reliability. The obtained stress versus fatigue cycle results were plotted as S-N curves. Fracture surface morphology was examined using a Quanta 200 environmental scanning electron microscope (Hillsboro, OR, USA).

### 2.5. Residual Stress Simulation

A thermo-mechanical coupling model for multi-layer welding of welded joints and a laser shock dynamic model (LSD) were established in ABAQUS (2022). The residual stress field obtained from the welding simulation is imported into the LSD model as the initial field, so as to simulate the effect of LSP on the residual stress of welded joints.

#### 2.5.1. Thermo-Mechanical Coupling Model for Multi-Layer Welding of Welded Joints

The weld model was established based on the actual weld groove geometry, employing eight-node thermo-mechanical coupled hexahedral elements (C3D8RT). The heat source model utilized was a double ellipsoidal heat source model [27], with corresponding heat source parameters and a three-pass welding sequence configured within the DFLUX subroutine. To balance computational accuracy and efficiency, the mesh size in the welding zone was set to 2 mm × 2 mm × 1 mm, while transition meshes were applied to other regions.

**Figure 4:**
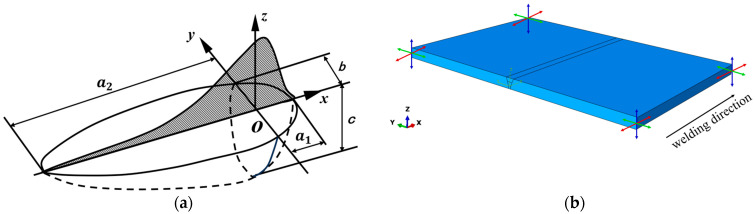
(**a**) Schematic diagram of Goldak’s double ellipsoid heat source (**b**) Welding finite element model.

The double ellipsoidal heat source model, proposed by Goldak, simulates the heat input generated by the welding arc. It incorporates many parameters such as welding speed, welding current, welding voltage, welding sequence, and starting position, offering high accuracy for simulating steel welding. The double ellipsoidal heat source model is illustrated in Figure 4a, where $a_{1}$ denotes the front semi-axial length along the welding direction of the double ellipsoidal heat source, $a_{2}$ denotes the rear semi-axial length along the welding direction, *b* signifies the semi-axial length in the weld width direction, *c* stands for the semi-axial length in the weld depth direction, *x* represents the distance from the heat source center along the welding direction, *y* indicates the distance from the heat source center in the melt width direction, and *z* refers to the distance from the heat source center in the melt depth direction. The heat source power distribution for the front and rear ellipsoids is determined by Equations (1) and (2). ∅ represents the input power, *η* denotes the welding efficiency, *U* is the welding voltage, and *I* is the welding current. Here, *η* = 0.75, *U* = 30 V, and *I* = 250 A. $f_{1}$ and $f_{2}$ represent the energy ratio of the front and rear ellipsoids, respectively, where $f_{1}+f_{2}=2$.

(1) $$q_{1}(x,y,z)=\frac{6\sqrt{3}f_{1}\emptyset }{a_{1}bc\pi \sqrt{\pi }}exp(-3\left(\frac{x^{2}}{{a_{1}}^{2}}+\frac{y^{2}}{b^{2}}+\frac{z^{2}}{c^{2}}\right))$$

(2) $$q_{2}(x,y,z)=\frac{6\sqrt{3}f_{2}\emptyset }{a_{2}bc\pi \sqrt{\pi }}exp(-3\left(\frac{x^{2}}{{a_{2}}^{2}}+\frac{y^{2}}{b^{2}}+\frac{z^{2}}{c^{2}}\right))$$

(3) ∅=η∗U∗I

(4) $$f_{1}=\frac{2a_{1}}{a_{1}+a_{2}}$$

(5) $$f_{2}=\frac{2a_{2}}{a_{1}+a_{2}}$$

The temperature-dependent parameters for the thermodynamic of DH36 material were obtained from the relevant reference [5]. The material parameters of the welding wire are assumed to be identical to those of the base material. During plate welding, fixtures were used to fix the plates. To ensure simulation deformation closely matches actual deformation while preventing rigid-body displacement of the welded plate, displacement constraints were applied only to the four vertical edges of the welded plate in the *x*, *y*, and *z* directions, as shown in Figure 4b. In the welding simulation, the middle root pass was welded first. After completion, a 50 s cooling step was applied. Subsequently, the front cover pass was performed, followed by another 50 s cooling period. Finally, the last back pass welding was performed, and a 1000 s cooling period was implemented at the end.

#### 2.5.2. Laser Shock Dynamic Model (LSD)

ABAQUS/Explicit (2022)was employed to simulate laser shock peening on the specimen. Since laser shock induces plastic deformation in materials at extremely high strain rates (exceeding 10^−6^), the Johnson–Cook constitutive model was adopted for explicit simulation [28,29]. Temperature variations remain negligible throughout processing. During the LSP process, a water confining layer and a black tape absorber layer always exist on the specimen surface, leading to insignificant temperature variation during the process, which has a minimal impact on material deformation and residual stress. Therefore, the influence of temperature on the simulation process was neglected. The simplified Johnson–Cook constitutive model is expressed as Equation (6).

(6) $$\sigma =\left(A+B\varepsilon^{n}\right)\left(1+C\left(\ln(\frac{\dot{\varepsilon }}{\dot{\varepsilon_{0}}})\right)\right)$$

where:

σ: the equivalent von Mises stress, in MPa
*A*: Initial yield stress of the material at the reference strain rate$\;\dot{\varepsilon_{0}}$ and reference temperature $T_{R}$
*B*, *n*: Strain hardening modulus and strain hardening index of the material at the reference strain rate $\dot{\varepsilon_{0}}$ and reference temperature $T_{R}$
ε: Effective plastic strain
$\dot{\varepsilon }$: Effective plastic strain rate
*C*: Strain rate strengthening parameter of the material

**Figure 5:**
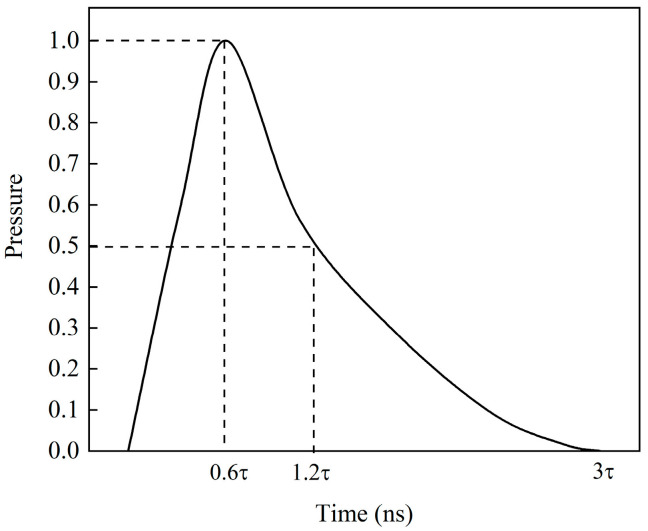
Distribution model of pressure wave time characteristics.

During the laser shock process, the laser shock wave generated by plasma acts on the metal surface and propagates inward continuously. When the peak pressure of the shock wave exceeds the dynamic yield limit of the material, plastic deformation occurs on the metal surface, inducing residual compressive stress. After the laser pulse ends, due to the presence of the confining layer, the plasma does not disappear immediately and continues to exert pressure, resulting in an impact pressure duration several times longer than the laser pulse duration. The shock wave loading curve is defined in the amplitude curve, and the duration of the shock wave pressure is generally three to four times the pulse width [24,30,31], with the amplitude curve shown in Figure 5. The peak shock pressure $P_{max}$ can be calculated using the Fabbro model [23], as expressed in Equations (7)–(9).

(7) $$P_{max}(GPa)=0.01\sqrt{\frac{\alpha }{2\alpha +3}}\sqrt{Z\left(g\cdot {cm}^{-2}\cdot s^{-1}\right)}\sqrt{I_{0}\left(GW\cdot {cm}^{-2}\right)}$$

(8) $$\frac{2}{Z}=\frac{1}{Z_{1}}+\frac{1}{Z_{2}}$$

(9) $$I_{0}\left(GW\cdot {cm}^{-2}\right)=\frac{4E\left(J\right)}{\pi D^{2}\left(cm\right)\tau \left(ns\right)}$$

where $I_{0}$ is the laser power density, calculated from Equation (9). In Equation (9), *E* is the laser energy, *D* is the spot diameter, and τ is the pulse width. α is the correction factor for plasma internal and thermal energy, α=0.25. *Z* is the acoustic impedance, with $Z_{1}$ and $Z_{2}$ being the acoustic impedances of the confining layer and the target material (impacted material), respectively. In the LSP process, deionized water is used as the confinement layer, and DH36 high-strength steel serves as the impacted material. Consequently, $Z_{1}$ is approximately $0.165\times 10^{6}\;g\cdot {cm}^{-2}\cdot s^{-1}$ and $Z_{2}$ is approximately $4.6\times 10^{6}\;g\cdot {cm}^{-2}\cdot s^{-1}$ [32]. Based on these parameters, Equation (7) simplifies to:

(10) $$P_{max}(GPa)=1.507\sqrt{I_{0}\left(GW\cdot {cm}^{-2}\right)}$$

The spatial distribution of the plasma-induced shock pressure *P* follows a Gaussian distribution [33]. The spatially and temporally inhomogeneous plasma pressure $P\left(r,t\right)$ can be determined by the following equation:

(11) $$P\left(r,t\right)=P\left(t\right)exp\left(-\frac{r^{2}}{2R^{2}}\right)$$

where $P\left(t\right)$ is the time-dependent function of the spatially uniform plasma pressure, whose value equals the product of the peak pressure $P_{max}$ and the shock loading curve. *r* denotes the radial distance from any point on the impact plane to the spot center, and *R* represents the spot radius. Based on the laser power density $I_{0}$ from Table 3, the peak pressure $P_{max}$ is calculated using Equation (10). $P\left(r,t\right)$ is defined in the VDLOAD subroutine, and the laser shock wave load is applied through the subroutine.

**Figure 6:**
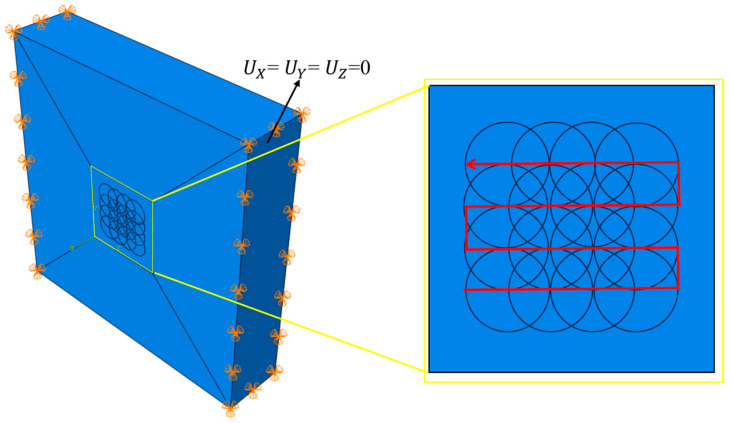
Geometric modeling of laser peening by FEA.

A three-dimensional solid finite element model was established. To simplify the model, a small weld specimen measuring 35 × 35 × 7.5 mm was adopted. To reduce computational load, a 4 × 4 mm impact array was applied only to the central 10 × 10 mm area in the middle of the specimen. The mesh was refined in the laser shock region with a mesh size of 0.15 mm and a thickness dimension of 0.1 mm, using C3D8R elements [34]. According to the actual clamping conditions, hinge constraints were applied to both side surfaces of the specimen, and laser shock simulation was performed on the upper and lower surfaces. The LSP finite element model is shown in Figure 6, where the yellow box area is the laser shock peening region, and the red arrow indicates the direction of the laser’s travel path.

## 3. Results and Discussion

### 3.1. Microhardness and Microstructure

**Figure 7:**
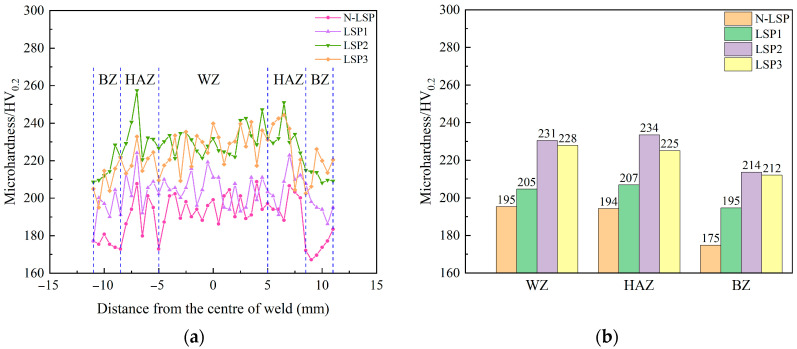
Surface microhardness in BZ, HAZ, and WZ of four welded joint specimen groups. (**a**) Hardness profile (**b**) Average hardness value.

**Figure 8:**
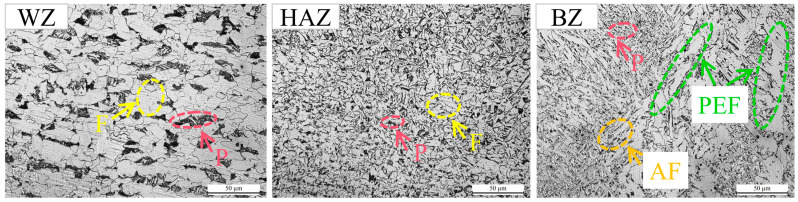
Metallographic microstructure of three different regions of N-LSP specimen.

**Figure 9:**
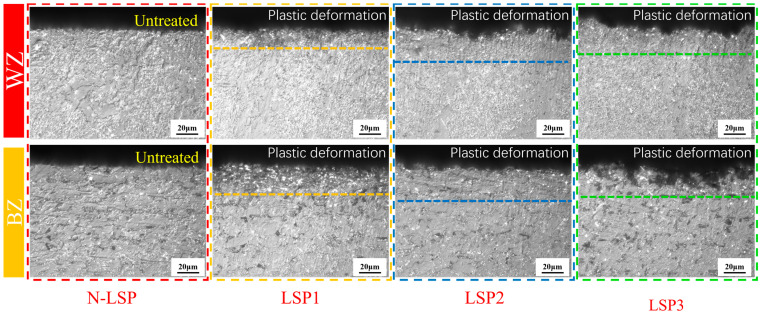
Schematic diagrams of the cross-sectional microstructures in the WZ and BZ of the four groups of specimens.

Figure 7 shows the surface microhardness distribution of four groups of specimens: N-LSP, LSP1, LSP2, and LSP3. Based on the microstructure and hardness distribution of the welded specimens, the welded joint can be divided into three zones: welding zone (WZ), heat affected zone (HAZ), and base zone (BZ). Figure 7a presents the hardness distribution across three regions for each group of welded specimens. Figure 7b presents the average hardness values across different regions for each group of welded specimens. Figure 8 shows the metallographic microstructures of the three zones in the N-LSP specimen (unstrengthened welded joint), while Figure 9 displays the cross-sectional metallographic microstructures of the four groups of specimens.

As observed in Figure 7, the four welded joint specimen groups exhibit similar microhardness distributions trends. The average hardness in the WZ and HAZ is comparable and higher than that in the BZ. Although the hardness in the WZ exhibits slight fluctuations, it remains relatively uniform overall. The hardness in the HAZ shows a trend of first increasing and then decreasing from the BZ to the weld center, with the highest microhardness value occurring in the HAZ. The hardness of different zones in the welded joint is primarily related to the microstructure within each zone. According to Figure 8, the microstructure of the BZ consists of polygonal ferrite (F) and pearlite (P) distributed between the ferrite grains. The HAZ undergoes recrystallization during welding, forming fine and uniform ferrite and pearlite, which leads to a notable increase in hardness. The WZ experiences high heat input and slow cooling, which allows directional solidification of the molten pool to form columnar grains. The overall microstructure in WZ exhibits a columnar grain structure, mainly composed of proeutectoid ferrite (PEF), acicular ferrite (AF), and pearlite. The hardness distribution and microstructural composition of each zone in the welded joint are consistent with the research results on DH36 welded joints by Gu et al. [35], both showing that the hardness of weld-related zones (WZ and HAZ) is higher than that of the base metal, with the maximum hardness occurring in the HAZ. Combined with the microstructures of the welded joint in Figure 8, the higher hardness of weld-related zones is attributed to the microstructural transformation of the weldment after the thermal cycle of welding, which forms a harder microstructure. The peak hardness in the HAZ is due to the fact that the HAZ undergoes multiple thermal cycles during welding, which is equivalent to a normalizing treatment and generates a more uniform and finer microstructure, resulting in relatively higher hardness in this region.

It can be observed in Figure 7 that after LSP, the surface microhardness of all regions in the specimens increased. The most significant improvement was seen in the LSP2 specimen: the WZ increased from 195 HV_0.2_ to 231 HV_0.2_, the HAZ increased from 194 HV_0.2_ to 234 HV_0.2_, while the BZ increased from 175 HV_0.2_ to 214 HV_0.2_. Compared to the unstrengthened state, surface microhardness increased by 18.5%, 20.6%, and 22.3%, respectively, across all regions. The surface microhardness of the WZ, HAZ, and BZ of the LSP1 specimen increased by 5.2%, 6.7%, and 11.4%, respectively, while that of the LSP3 specimen in each corresponding zone increased by 16.9%, 16%, and 21.2%, respectively. By comparing the surface microhardness of the three LSP specimen groups, it can be observed that when the laser energy increases from 5 J (LSP1) to 7 J (LSP2), the surface microhardness of the material rises. However, when energy reaches 9 J (LSP3), the surface microhardness no longer increases and instead slightly decreases. From Figure 9, it can be seen that the microstructure type of the welded joint does not change distinctly after LSP. However, compared with the original untreated specimen (N-LSP), LSP1, LSP2, and LSP3 exhibit varying degrees of plastic deformation—the thickness of the plastic deformation layer is the largest for LSP2 (7 J), followed by LSP3 (9 J), and the smallest for LSP1 (5 J). The plastic deformation of each group by LSP in Figure 8 is consistent with the hardness results in Figure 6. Combining the EBSD analysis results of DH36 welded joints by Chi et al. [36] and TC6 titanium alloy by Li et al. [37], it can be concluded that the mechanism underlying LSP’s enhancement of microhardness primarily involves dislocation strengthening and grain refinement. When the laser impacts the material surface, the immense shock wave pressure induces plastic deformation, leading to the generation and accumulation of high-density dislocations. Multiplied dislocations further hinder the movement of dislocations, significantly enhancing the material’s resistance to deformation and achieving strengthening effects [38]. This shock-induced plastic deformation promotes fragmentation and recrystallization of coarse grains, resulting in grain refinement and thereby enhancing hardness.

When the laser energy increases from 5 J to 7 J, the peak pressure $P_{max}$ induced by the laser shock wave rises from 2.8342 GPa to 3.3534 GPa. The increased shock wave energy acting on the specimen surface induces more pronounced plastic deformation. This leads to substantial dislocation multiplication and the formation of entangled structures, further hindering dislocation movement, and enhancing grain refinement. Thus, the hardness at 7 J shows a greater increase compared to that at 5 J. Liu et al. [39] also observed that higher laser energy yields more remarkable strengthening effects and greater increases in microhardness. When the energy reaches 9 J, the $P_{max}$ of 3.8024 GPa exceeds the material’s energy absorption threshold. At this point, the material surface can no longer achieve further dislocation multiplication or grain refinement, so the hardness no longer increases. Due to the energy saturation at 9 J, the surface grains undergo slight coarsening caused by local overheating, resulting in a mild decrease in hardness compared to 7 J. Montross et al. [40] and Peyre et al. [41] also found that when laser energy overpasses the material threshold, overheating triggers dislocation annihilation and grain melting–recrystallization. This leads to stress relaxation, causing hardness to cease increasing or even decrease.

### 3.2. Surface Morphology

**Figure 10:**
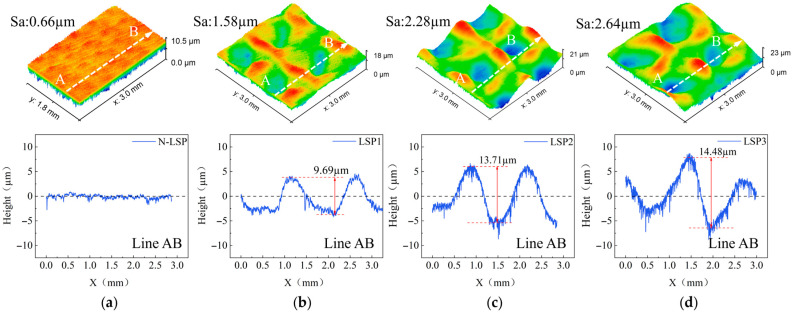
Surface morphology of welded joints with different LSP processing parameters (**a**) N-LSP, (**b**) LSP1, (**c**) LSP2, (**d**) LSP3.

The surface morphology of the DH36 welded joint specimens before and after LSP is shown in Figure 10. Excessively high surface roughness tends to cause stress concentration, leading to fatigue crack initiation and significantly reducing the fatigue life of the specimens. For the original tensile-compression fatigue specimens, the weld reinforcement was ground off, and the surface was polished with sandpaper to ensure a low surface roughness. This avoids stress concentration and subsequent fracture caused by machining scratches. To investigate the comprehensive effect of increased surface roughness induced by LSP on fatigue performance, no surface treatment was performed on the specimens after LSP. As shown in Figure 10, the surface roughness values Sa for the N-LSP, LSP1, LSP2, and LSP3 specimen groups were 0.66 μm, 1.58 μm 2.28 μm, 2.64 μm, respectively. After LSP, specimen surface roughness increased significantly, rising by 139.4%, 245.5%, and 300% compared to the original smooth surface. Increased surface roughness tended to induce fatigue cracks on the surface. However, the surface roughness values Sa after LSP remained below 3.2 μm, with no visible machining traces on the specimen surface, which prevented rapid fracture due to apparent stress concentration. The increase in surface roughness of the specimens after LSP occurs because the uneven energy distribution within the circular laser spot causes non-uniform plastic deformation upon impact, resulting in pits and protrusions on the surface that elevate roughness. Meanwhile, the immense energy from the laser shock wave induces plastic deformation in the material surface. Plastic deformation parallel to the material surface cannot absorb all impact energy within the extremely short laser pulse duration. The remaining impact energy is released through plastic deformation perpendicular to the material surface. As laser energy increases from 5 J to 9 J, peak pressure continues to rise. More energy fails to be absorbed by the surface and is converted into plastic deformation on the material surface. The resulting extensive surface undulations cause continuous increases in surface roughness and pit depth. When energy reaches 9 J, the plastic deformation generated on the surface no longer increases significantly, thereby slowing down the rate of roughness increase [42].

### 3.3. High Cycle Fatigue Performance

**Figure 11:**
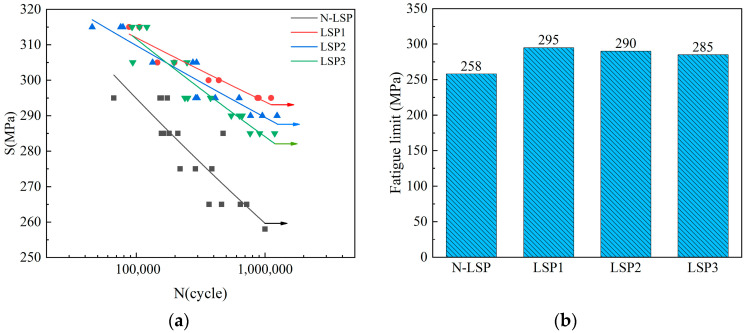
(**a**) S-N curves for different specimens (**b**) Fatigue limits for different specimens.

The vertical axis S of Figure 11 represents stress levels and the horizontal axis N denotes the number of cycles. As shown in Figure 11, the fatigue limit of laser-strengthened specimens increased compared to the unstrengthened specimens. Among them, the LSP1 specimen exhibited the highest fatigue limit of 295 MPa, representing a 14.34% improvement over the 258 MPa fatigue limit of the N-LSP specimen. The improvement effect of the LSP2 specimen was slightly lower than that of the LSP1 specimen, with an increase of 12.4%, and the LSP3 specimen had the lowest improvement range, with an increase of 10.47%. Under high stress conditions of 295 MPa, the average fatigue life of the LSP1, LSP2, and LSP3 specimens increased by 5.99 times, 1.96 times, and 1.1 times, respectively, compared to the original average fatigue life. Tensile-compression tests revealed that the fatigue limit of specimens reached its highest value at a laser energy of 5 J, differing from the previous view that 7 J was the optimal impact energy. Fracture morphology was observed to analyze the fracture mechanism and explore the strengthening mechanism of LSP on tensile-compression fatigue specimens.

**Figure 12:**
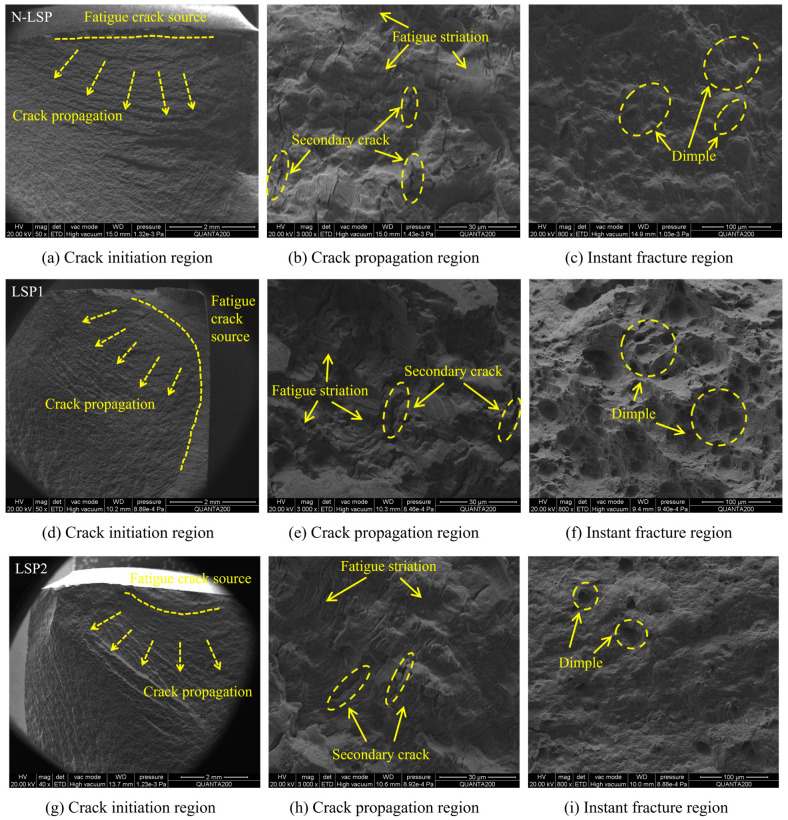

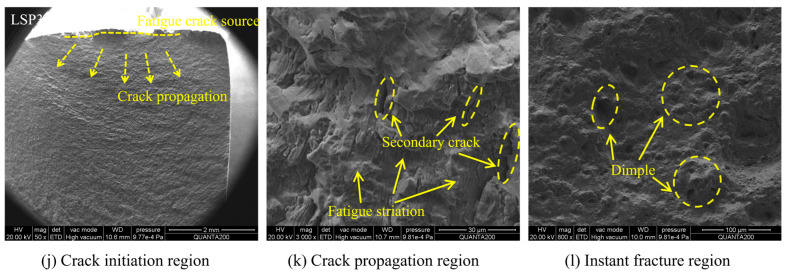
Fracture morphology of Different Samples: (**a**–**c**) N-LSP, (**d**–**f**) LSP1, (**g**–**i**) LSP2, (**j**–**l**) LSP3.

Fatigue fracture surfaces can generally be divided into three zones: the crack initiation zone, the crack propagation zone, and the instantaneous fracture zone. Experimental results showed that each specimen fractured in the HAZ. This is attributed to the large abrupt hardness change and uneven degree of grain refinement at the interface between the HAZ and the BZ. Fatigue crack origins in N-LSP specimens initiate at the surface, which exhibits relatively rough texture (Figure 12a). In contrast, the crack propagation zone of specimens strengthened by LSP is relatively flat and smooth. The fracture edge area of the LSP1 specimen exhibits a smooth polished region resulting from compression and friction at the crack surface. Based on the extent of the polished zones and the crack propagation direction, it is estimated that cracks in the LSP1 specimen primarily emerge from subsurface. Their crack initiation zones are smooth with extensive flat areas, where several crack origins converge to form steps, presenting a ratchet-like pattern (Figure 12d). LSP2 specimen exhibits a steep gradient between surface and internal hardness, resulting in more uneven hardness distribution. Multiple crack initiation sources emerge, with the initiation zone located in the surface weak zone and a reduced flat area (Figure 12g). Specimen LSP3 has higher surface roughness, with cracks initiating on the surface in a rough and uneven initiation zone (Figure 12j).

The N-LSP specimen shows a fast crack propagation, large fatigue striation spacing, and numerous secondary cracks causing localized fracture of the fatigue striations (Figure 12b). The LSP1 specimen displays dense and uniform fatigue striations in the crack propagation zone, with fewer and finer secondary cracks (Figure 12e). LSP induces residual compressive stress in the material surface, significantly retarding crack propagation. LSP causes the material to undergo severe plastic deformation in a short time. High strain rate deformation forms deformation twins on the surface, which impede dislocation slip, leading to an increase in dislocation density and further hindering crack propagation. The high surface hardness of the LSP2 specimen hinders fatigue crack propagation, resulting in small-spaced fatigue striations (Figure 12h). The LSP3 specimen has increased spacing between fatigue striations and a higher number of secondary cracks. There is smooth, plate-like region without fatigue striations (Figure 12k), which impedes normal crack propagation. This leads to fractures and distortions in some fatigue striations, causing uneven distribution of fatigue striations.

The instantaneous fracture zone of the N-LSP specimen primarily consists of dense, small dimples, and the overall fracture surface is relatively uniformly rough (Figure 12c), indicating ductile plastic fracture behavior. The instantaneous fracture zone of the LSP1 specimen exhibits numerous large and deep dimples. The fracture surface is covered with large honeycomb-like pits caused by severe plastic deformation (Figure 12f), reflecting the characteristics of high-ductility fracture. LSP refines the weld joint microstructure and enhances material plasticity. Due to sufficient plastic reserves in the instantaneous fracture zone, microvoids can fully nucleate, grow, and coalesce, forming a large number of deep ductile pits and resulting in high-ductility instantaneous fracture [43]. Specimens LSP2 and LSP3 primarily exhibit elongated dimples (Figure 12i,l), indicating decreased plastic deformation capacity. However, these dimples are larger and deeper than those in the original specimen, which shows that LSP enhances the material’s plastic deformation capability, enabling the growth and coalescence of microvoids.

LSP refines surface grains, and this grain refinement impedes crack propagation, causing cracks to originate primarily subsurface. It also induces compressive stress in the surface layer, which delays crack initiation. For the LSP1 specimen, the stress concentration effect caused by increased surface roughness is not significant, and the positive effects of LSP are more prominent. Under laser treatment, the microstructure within the material undergoes a certain degree of refinement. Dislocation multiplication and residual compressive stress hinder the early rapid propagation of cracks. The fatigue striations in the propagation zone are fine and dense, and the material has more time and space for plastic deformation before fracture, allowing microvoids to fully nucleate, grow, and coalesce. As a result, a large number of deep dimples appear in the instantaneous fracture zone [43]. For LSP2 and LSP3 specimens, surface roughness is further increased compared with LSP1. Enhanced stress concentration effects from microscopic surface protrusions and pits accelerate crack propagation rates. The time available for micro-pores to fully nucleate and grow is shortened, resulting in fewer and shallower toughening pits [44]. The LSP2 specimen has the most significant increase in surface microhardness, which enhances the material’s strength and deformation resistance. High hardness hinders rapid crack propagation, thereby improving fatigue performance [45]. For LSP3 specimens, the excessive energy of 9 J may cause localized overheating of the material surface, leading to grain coarsening and even microcrack formation. Simultaneously, the excessive impact force may result in uneven residual compressive stress distribution inside the material, thereby degrading its fatigue performance [46].

In conclusion, the LSP1 specimen achieves optimal fatigue performance through the synergistic effects of relatively low surface roughness, LSP-induced residual compressive stress, grain refinement, and dislocation multiplication. The LSP2 specimen exhibits higher residual stress and surface microhardness, but its rougher surface resulted in fatigue performance inferior to that of the LSP1 specimen. The LSP3 specimen has excessively high energy input exceeding the material’s tolerance threshold, coupled with increased surface roughness. Consequently, its fatigue performance is inferior to both LSP1 and LSP2 specimens, though it still outperforms the original specimen. The tensile-compression fatigue test results confirm that the optimal laser power density for DH36 welded joints is 3.54 GW/cm^2^.

### 3.4. Residual Stress Modification

**Figure 13:**
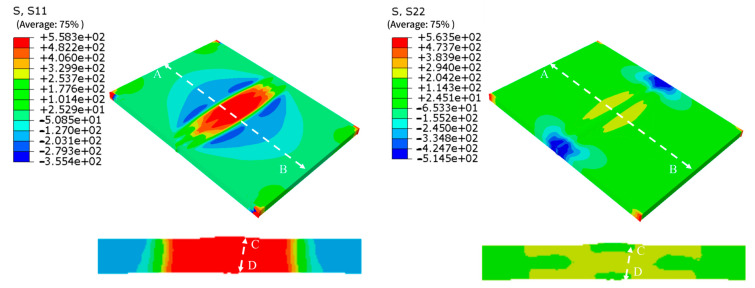
Residual stress contour plots. (**a**) longitudinal stress (**b**) transverse stress.

**Figure 14:**
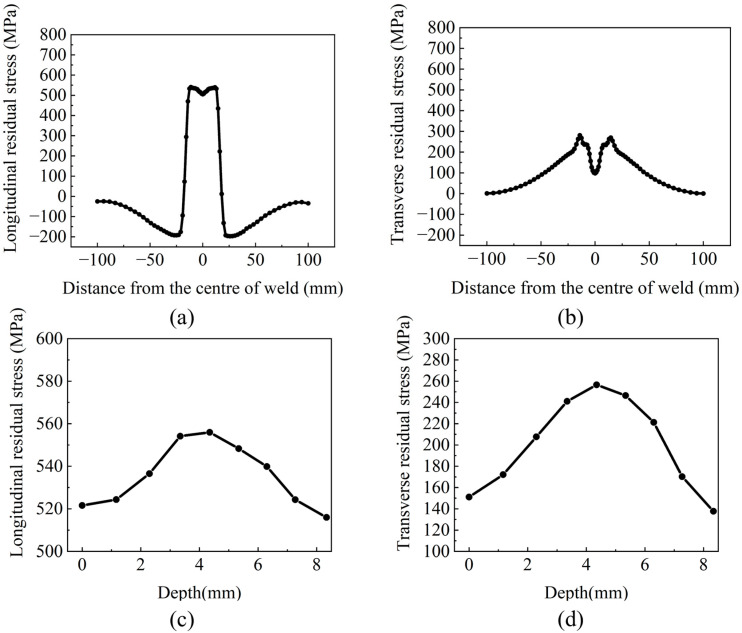
Residual stress distribution. (**a**) longitudinal stress along Line AB (**b**) transverse stress along Line AB (**c**) longitudinal stress along Line CD (**d**) transverse stress along Line CD.

The residual stress contour map obtained from the welding simulation is shown in Figure 13. Based on this contour map, the longitudinal and transverse residual stress along the centerline AB (on the midline of the specimen’s upper surface) and along the line CD (in the central cross-section of the specimen) were plotted, as shown in Figure 14.

As shown in Figure 13, the longitudinal residual stress and transverse residual stress are distributed symmetrically along the weld, with the longitudinal residual stress being significantly greater than the transverse residual stress. Figure 11b and Figure 14a reveal that the residual stress distribution roughly exhibits a “W” shape. The tensile stress induced by welding is primarily concentrated in the weld and within 23 mm from the weld center, while the residual stress decreases slightly at the weld center. This occurs because the metal at the weld center can undergo free deformation during solidification, whereas the metal at the interface with the base metal is constrained by the base metal during the welding flow process.

As shown in Figure 11a, the longitudinal residual stress in the weld region is observed to be around 530 MPa, exceeding the yield strength of the base metal. At a distance of about 23 mm from the weld center, the residual stress rapidly decreases, transforming into compressive stress of around 200 MPa. The residual compressive stress diminishes as the distance from the weld center increases, eventually approaching zero. Similar findings were reported in the paper by Ding et al. [47]. This phenomenon occurs because the weld metal melts during welding. During cooling, due to the volume shrinkage from liquid to solid and the rigid constraints of the unmelted base metal on both sides, significant residual tensile stress is generated in the WZ. Materials farther from the weld center do not undergo complete melting during welding; their shrinkage during cooling is between that of the WZ and the adjacent BZ. Subjected to the pulling of the weld and the squeezing of the base metal, the superposition of these two effects converts the residual stress from tensile to compressive. The farther from the weld center, the less thermal influence the material receives, and the material can release residual stress through its own elastic deformation. Consequently, the residual compressive stress gradually decreases to zero as the distance increases [48]. According to Figure 11b, transverse residual stress reaches a maximum of 280 MPa on both sides of the weld. The farther from the weld center, the smaller the residual tensile stress, and the transverse residual stress decreases to zero in the BZ. Figure 14c,d reveal that both longitudinal and transverse residual stress exhibit maximum values at the mid-thickness position. This is because the middle layer serves as the root pass, constrained by the subsequent cover layers, upper and lower. Residual tensile stress induced by welding significantly reduces the fatigue life of welded structural components. Therefore, LSP treatment is employed to weaken tensile stress and mitigate its adverse effects. Considering that cracks typically initiate on the surface and to simplify the model, the maximum surface longitudinal residual stress of 540 MPa and the maximum surface transverse residual stress of 280 MPa are taken as the initial stress field for the laser shock post-processing (LSP) model.

**Figure 15:**
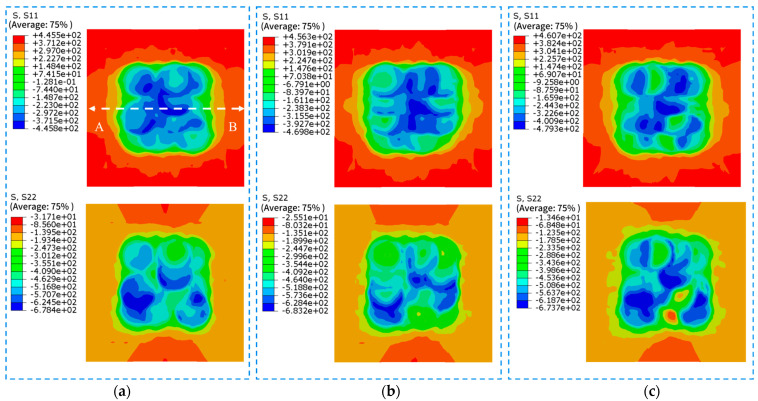
Distribution of residual stress on the surface of the three sample sets. (**a**) LSP1 (**b**) LSP2 (**c**) LSP3.

**Figure 16:**
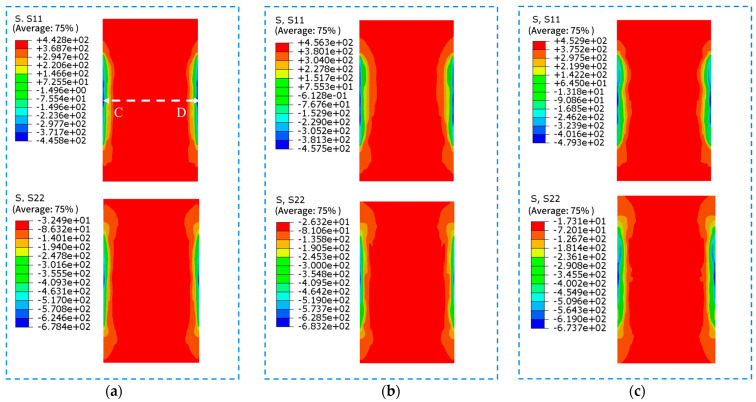
Distribution of residual stress in depth of the three sample sets. (**a**) LSP1 (**b**) LSP2 (**c**) LSP3.

**Figure 17:**
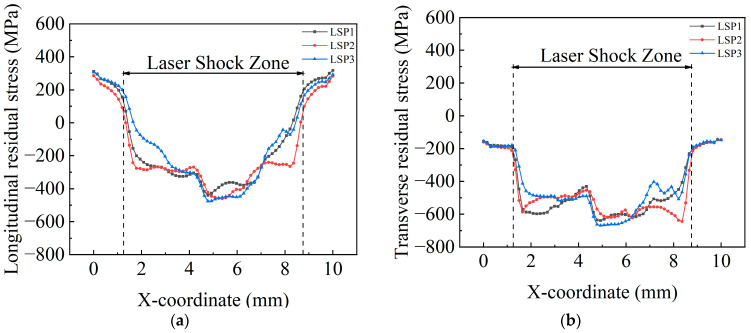
Distribution of residual stress on the surface along Line AB. (**a**) longitudinal stress (**b**) transverse stress.

**Figure 18:**
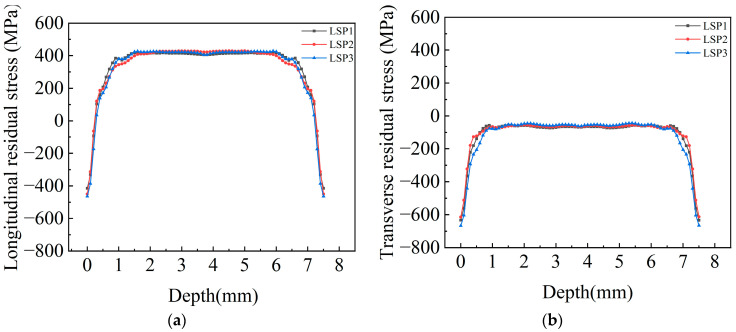
Distribution of residual stress in depth along Line CD. (**a**) longitudinal stress (**b**) transverse stress.

LSP simulations with different peak pressures are performed on the LSP model subjected with the applied initial stress field. The obtained residual stress contour maps of the surface and cross-section are shown in Figure 15 and Figure 16, respectively. Residual stress curves at the middle position of each contour map are depicted in Figure 17 and Figure 18.

As can be seen in Figure 15, the welding residual tensile stress on the surface of the strengthened area of the specimens is converted into residual compressive stress after LSP, and the transverse residual compressive stress is numerically larger than the longitudinal residual compressive stress. Additionally, the residual tensile stress in the unstrengthened regions also decreases to some extent. The residual stress distribution is non-uniform, exhibiting “residual stress holes.” The distribution uniformity of transverse residual stress is worse than that of longitudinal residual stress. Specimen LSP3 exhibits the most uneven longitudinal residual stress distribution. With the increase in energy, transverse residual tensile stress is generated in the strengthened area.

Figure 16 reveals that a layer of residual compressive stress forms at a certain depth after LSP. Significant longitudinal residual tensile stress persists in the depth direction, while transverse residual stress is nearly zero. The residual stress distributions across the cross-sections of the three specimen groups show little variation.

Figure 17 shows the residual stress curves on the specimen surfaces. It can be observed that the transverse and longitudinal residual stress of the three specimen groups do not differ significantly, with the transverse residual compressive stress being greater than the longitudinal residual stress. The distribution of transverse residual stress is more uniform in the strengthened area. The maximum longitudinal compressive stress for LSP1, LSP2, and LSP3 specimens is −440.364 MPa, −460.818 MPa, and −475.811 MPa, respectively. The maximum transverse residual compressive stress is −638.77 MPa, −644.48 MPa, and −668.988 MPa, respectively. It can be observed that as the laser energy increases, the residual compressive stress increases slightly, and the magnitude of the increase is very small. The residual stress curve of the LSP2 specimen exhibits the flattest profile in the strengthened area, while the LSP3 specimen shows the worst flatness. After LSP, the welding-induced residual tensile stress in the overlap area is completely neutralized. The average value of longitudinal residual stress is −339 MPa, and the average value of transverse residual stress is −554 MPa. The absolute stress values correspond to 62.78% and 197.86% of the original welding-induced residual tensile stress, respectively.

Figure 18 demonstrates that LSP significantly reduces residual tensile stress in the depth direction. The three specimen groups exhibit nearly identical effects in this dimension. The depth of longitudinal residual compressive stress is approximately 0.2 mm. The longitudinal residual tensile stress inside the welded joint after LSP is around 415 MPa, representing a 23.15% reduction compared to the original 540 MPa longitudinal residual tensile stress. Throughout the entire depth, residual tensile stress in the transverse direction is offset, forming residual compressive stress of about −50 MPa in the central region. The absolute value of this compressive stress accounts for 17.86% of the original 280 MPa weld residual tensile stress.

Based on the residual stress data obtained from the above simulations, it can be inferred that the material achieves optimal power density at 5 J. Further increasing the energy does not significantly increase the residual compressive stress. The residual stress results are consistent with tensile-compression fatigue test outcomes. The specimen has reached the energy threshold at 5 J, exhibiting residual tensile stress comparable to those at 7 J and 9 J while maintaining the lowest surface roughness. Consequently, its tensile-compression fatigue limit significantly surpasses that of the other two groups.

### 3.5. Discussion

According to the above discussion, it can be seen that the laser energy corresponding to the maximum microhardness is 7 J, while the energy for the optimal fatigue performance is 5 J. There is a difference in the energies of highest microhardness and best fatigue performance.

The increase in microhardness is mainly determined by the degree of plastic deformation of the surface layer. When the laser energy is 7 J, compared with 5 J, the surface of the specimen receives a larger shock wave energy, the surface produces a more significant plastic deformation, resulting in increased degree of dislocation multiplication and grain refinement, which significantly improves the hardness. When the energy reaches 9 J, the shock wave energy acting on the specimen surface hits the material’s energy absorption threshold, the surface layer can no longer achieve further grain refinement or dislocation multiplication. As a result, the 9 J specimen hardness does not increase compared to that with 7 J, and even because the local overheating led to a slight grain coarsening, the hardness is slightly decreased. Therefore, the specimen surface attains the maximum microhardness under the energy of 7 J laser energy.

The fatigue performance is influenced by the synergistic effects of surface roughness, crack initiation mechanisms and residual stresses. At the energy of 5 J, the microhardness of the specimen surface has been significantly improved compared to the original untreated specimen, which can effectively slow down surface crack initiation and expansion. Among the three groups of laser energies, the surface roughness of the 5 J specimen is 1.58 μm, which is considerably lower than the surface roughness of 7 J (2.28 μm) and 9 J (2.64 μm). Its surface is relatively flat, resulting in a weak stress concentration effect that effectively suppresses surface cracks; cracks instead initiate in the subsurface (Figure 12d). Although the 7 J specimen exhibits the largest surface microhardness, its surface has deeper pits and higher bumps compared to the 5 J specimen. These geometrical defects produce serious stress concentration, leading to surface crack initiation (Figure 12g). Once the cracks sprout on the surface, they will expand rapidly under cyclic loading and directly shorten the fatigue life.

From the simulation results in Section 3.4, it can be seen that the maximum longitudinal residual stresses of the 5 J, 7 J and 9 J specimens are −440.36 MPa, −460.82MPa, −475.81 MPa, and the maximum transverse residual compressive stresses are −638.77 MPa, −644.48 MPa, −668.988 MPa, the difference among the three groups is only 5–8%, and their effects in the depth direction are nearly identical. The 5 J specimen already forms a 0.2 mm-thick longitudinal residual compressive stress layer in the depth direction, which can effectively impede the crack extension.

Although the 7 J specimen has the highest surface microhardness and a slight increase in residual compressive stress compared to the 5 J specimen, its surface roughness is 44.3% higher than that of the 5J specimen. In contrast, the 5 J specimen, with residual compressive stress comparable to the 7 J specimen, achieves the best fatigue performance by combining relatively high surface microhardness and the minimum surface roughness (its fatigue limit is 295 MPa, an increase of 14.34% compared to the original specimen).

## 4. Conclusions

This study addresses the engineering challenge of fatigue life enhancement for DH36 welded joints in deep-sea service by applying LSP technology to reinforce weld fatigue. It investigates the effects and mechanisms of three different LSP energy levels (5 J, 7 J, 9 J) on surface microhardness, surface morphology, fatigue life, and residual stress in DH36 welded joints, filling the gap in engineering applications.

This paper first employs the Fabbro model to determine the plastic deformation saturation range for LSP of DH36 welded joints as 8.86 J–11 J (with spot diameter 3 mm, overlap ratio 50%, pulse width 20 ns). Measured hardness data confirm the most effective hardness improvement occurs at 7 J, while surface hardness decreases at 9 J due to grain coarsening from local overheating. Tensile-compression fatigue tests were conducted on three groups of specimens with different energy levels. Comparative analysis shows that 5 J delivers optimal fatigue performance, indicating that LSP parameters should not be determined solely based on hardness. Simulations of welding and LSP processes accurately quantify residual stress distributions of specimens after welding and LSP, enhancing engineering reliability. Simulations revealed that LSP effectively neutralizes residual compressive stresses from welding, the specimens already achieve relatively ideal residual compressive stress effects at the energy of 5 J. The main conclusions are as follows:

1. LSP significantly enhances the surface microhardness of specimens, with the most pronounced increase observed at 7 J energy. The WZ hardness rises from 195 HV_0.2_ to 231 HV_0.2_, the HAZ from 194 HV_0.2_ to 234 HV_0.2_, while the BZ increases from 175 HV_0.2_ to 214 HV_0.2_. Compared to the unhardened state, surface microhardness increases by 18.5%, 20.6%, and 22.3% in these regions, respectively. When laser energy reaches 9 J, it has exceeded the material threshold; as a result, the material no longer undergoes further dislocation multiplication or grain refinement, and the surface microhardness of the specimen ceases to increase.
2. Surface roughness increases with the increase in laser energy. The surface roughness values for the N-LSP, LSP1, LSP2, and LSP3 groups were 0.66 μm, 1.58 μm, 2.28 μm, 2.64 μm, respectively. To ensure low surface roughness, laser energy should not be excessively high.
3. LSP can delay the initiation and propagation of surface cracks, reduce the spacing of fatigue striations, form deeper and larger dimples, and thus improve the fatigue performance. After LSP, the fatigue limits of the specimens are all increased: the fatigue limits of LSP1, LSP2, and LSP3 specimens are 295 MPa, 290 MPa, and 285 MPa, respectively, representing improvements of 14.34%, 12.4%, and 10.47% compared to the 258 MPa fatigue limit of the original N-LSP specimen.
4. The simulation of welding and LSP on DH36 material demonstrates that LSP can significantly reduce residual tensile stress. DH36 welded joints exhibit large residual tensile stress after welding, with maximum longitudinal residual tensile stress of 540 MPa and maximum transverse residual stress of 280 MPa. After LSP strengthening, the residual tensile stress in the weld is eliminated, and residual compressive stress is introduced on the strengthened surface. In the depth direction, the thickness of the longitudinal residual compressive stress is 0.2 mm. The longitudinal residual tensile stress within the weld joint is approximately 415 MPa, representing a 23.15% reduction compared to the original 540 MPa longitudinal residual tensile stress in the weld. Transverse residual tensile stresses are completely neutralized in the thickness direction and converted to compressive stresses of approximately −50 MPa.

## Figures and Tables

**Table 1:** Chemical composition of the DH36 steel and YCJ501-1 filler metal (in wt.%).

Materials	C	Si	Mn	P	S	Cr	Ni	Cu	Mo	V
DH36	0.141	0.243	1.54	0.0128	0.0033	0.0275	0.009	0.0131	0.003	0.0034
YCJ501-1	0.054	0.36	1.42	0.019	0.009	0.02	0.05	0.01	0.01	0.003

**Table 2:** The mechanical properties of DH36 steel.

Material	Yield Strength σ0.2 [MPa]	Ultimate Tensile Strength σ_b_ [MPa]	Elongation δ (%)
DH36	413	570	29.47

**Table 3:** Parameters of LSP treatments.

Specimen	Lapping Rate	Spot Diameter (mm)	Energy (J)	Power Density (GW/cm^2^)	Laser Impact
LSP1	50%	3	5	3.54	1
LSP2	50%	3	7	4.95	1
LSP3	50%	3	9	6.37	1

## Data Availability

The original contributions presented in this study are included in the article. Further inquiries can be directed to the corresponding author.
